# Serum Biomarker-Based Diagnostic Tools for Primary Hyperparathyroidism: A Systematic Review and Meta-Analysis with Implications for Primary Care

**DOI:** 10.3390/healthcare14081001

**Published:** 2026-04-10

**Authors:** Yelson Alejandro Picón-Jaimes, Judit Mauri Juliachs, Iván Arrufat Martin, Milena Lopez-Castaño

**Affiliations:** 1 Centre d’Atenció Primària La Pau, Institut Català de la Salut, 08007 Barcelona, Spain; 2 Vice-Rectorate for Graduate Studies, Research and Social Protection, Corporación Universitaria del Meta, Villavicencio 510001, Colombia; juditmauri.bcn.ics@gencat.cat (J.M.J.); ivanarrufat.bcn.ics@gencat.cat (I.A.M.); milelopez77@gmail.com (M.L.-C.)

**Keywords:** hyperparathyroidism, primary, parathyroid hormone, calcium, phosphorus, biomarkers, sensitivity and specificity, ROC Curve

## Abstract

**Highlights:**

**What are the main findings?**

- The calcium–phosphorus ratio (Ca/P) and the Parathyroid Function Index (PFindex) showed the highest diagnostic accuracy for identifying primary hyperparathyroidism, with pooled sensitivities and specificities above 90% in the meta-analysis.
- Among the evaluated tools, the Ca/P ratio emerged as the most widely studied and accessible screening index, while PFindex demonstrated the best discriminatory capacity when directly compared with other indices.

**What are the implications of the main findings?**

- Simple biochemical indices derived from routine laboratory tests may facilitate earlier detection of primary hyperparathyroidism, particularly in primary care or resource-limited settings.
- These indices should complement rather than replace clinical assessment and standard biochemical evaluation, and further prospective studies are needed to validate emerging indices such as Ca × Cl/P and dynamic tests.

**Abstract:**

**Background:** Hyperparathyroidism is a common endocrine disorder, and its diagnosis can be complex. Various indices based on blood biomarkers have been proposed to improve diagnostic accuracy. The objective of this systematic review was to analyze the diagnostic utility of different indices in primary hyperparathyroidism. **Methods:** A systematic review was performed with searches up to January 2026. Risk of bias was assessed, and a meta-analysis was conducted for indices with two or more studies, calculating sensitivity, specificity, and other accuracy measures. The certainty of the evidence was evaluated using the GRADE system. **Results:** Twelve studies were included. The calcium–phosphorus ratio demonstrated a sensitivity of 91.6%, specificity of 89.3%, and an area under the curve of 0.957. The parathyroid function index showed a sensitivity of 94.4% and specificity of 94.2%; however, this finding is based on only two studies and requires validation in larger cohorts. The Wisconsin index also showed good performance. Other indices, including the Ca × Cl/P ratio (evaluated in a single study), yielded promising results but with very limited evidence that precludes firm conclusions. All indices performed poorly in cases with normal calcium. Certainty assessment indicated moderate evidence for the main indices and low or very low evidence for the others. **Conclusions:** The calcium–phosphorus ratio and the parathyroid function index are valid and useful tools for the diagnosis of primary hyperparathyroidism, with excellent performance. The calcium–phosphorus ratio is especially valuable due to its simplicity and accessibility for screening. No index should be used in isolation; integration with clinical evaluation remains essential.

## 1. Introduction

Hyperparathyroidism is one of the most prevalent endocrinopathies in clinical practice. Its incidence is comparable between men and women under 45 years of age but increases notably in women from that age onward [1,2]. Prevalence is higher in people of African descent, followed by the white population, and is lower in Asian and Hispanic individuals. Clinical manifestations derive fundamentally from alterations in calcium-phosphorus metabolism, notably hypercalcemia, bone tissue compromise, and renal complications [1,3].

Primary hyperparathyroidism (PHPT) originates from autonomous and inappropriate secretion of parathyroid hormone (PTH), usually secondary to a parathyroid adenoma. This hormonal excess causes dysregulation of calcium homeostasis [1,4]. At the bone level, PTH stimulates resorption, releasing calcium and phosphorus into the bloodstream; in the kidney, it increases tubular reabsorption of calcium and promotes phosphorus excretion, in addition to inducing calcitriol synthesis, which in turn enhances intestinal calcium absorption [1,2,4]. A central pathophysiological element is the alteration of the set point of the calcium-sensing receptor (CaSR) in the affected gland, which prevents adequate suppression of PTH despite elevated serum calcium concentrations [5,6,7].

For its part, secondary hyperparathyroidism (SHPT) represents a compensatory response to hypocalcemia or vitamin D deficiency, being especially frequent in chronic kidney disease and intestinal malabsorption syndromes [8,9,10]. When this stimulation persists over time, tertiary hyperparathyroidism (THPT) may develop, characterized by functional autonomy of the glands and sustained PTH secretion with hypercalcemia, even after correcting the initial stimulus [11,12,13]. Other less common entities include normocalcemic hyperparathyroidism (NHPT), defined by normal calcium levels with persistent PTH elevation after excluding secondary causes, and ectopic or paraneoplastic hyperparathyroidism, associated with PTH-producing tumors or related peptides [14,15,16,17].

Among these entities, NHPT represents a particularly challenging diagnostic scenario. The distinction between NHPT and SHPT is one of the most critical issues in this field, as both conditions present with elevated PTH and normal calcium levels, leading to significant biochemical overlap. Secondary causes for increased PTH concentration, including vitamin D deficiency, malabsorption syndromes, renal insufficiency, primary hypercalciuria, and use of medications such as lithium or thiazide diuretics, must be rigorously excluded before establishing a diagnosis of NHPT. This diagnostic uncertainty has direct clinical implications, as misclassification may lead to inappropriate surgical referrals or delayed treatment of correctable secondary causes [17,18].

Diagnosis is based on the integration of biochemical parameters such as serum calcium, phosphorus, and PTH, together with clinical evaluation. However, in situations such as NHPT, interpretation is complex due to overlap with other metabolic conditions, such as vitamin D deficiency or chronic renal insufficiency [18,19,20,21].

In this context, various biochemical indices and dynamic tests have been proposed to improve diagnostic discrimination between the different forms of hyperparathyroidism. The calcium/phosphorus ratio (Ca/P) represents the simplest and most accessible index, requiring only routine biochemical parameters available in any clinical laboratory, making it particularly suitable for primary care settings where PTH measurement may not be immediately available or cost-effective [22,23,24]. The Parathyroid Function Index (PFindex: Ca × PTH/P) incorporates PTH into its formula, potentially offering superior discrimination between primary and secondary hyperparathyroidism by more directly reflecting autonomous parathyroid function. The Wisconsin Index (WIN: Ca × PTH), originally designed to predict multiglandular disease during parathyroidectomy, has been explored as a diagnostic tool with variable results [22,23,24,25]. More recently, the Ca × Cl/P quotient has emerged as a promising index that may offer improved performance in normocalcemic presentations. Also, the chloride/phosphorus ratio (Cl/P) and the Thiazide Challenge Test (TCT) represent additional approaches with more limited evidence. These indices are particularly relevant for primary care, where they can help reduce the diagnostic gap in patients with suspected parathyroid disorders, better focus treatments, and guide appropriate referrals to specialized care. However, despite promising individual study results, methodological and population heterogeneity prevents establishing firm conclusions about their comparative clinical utility, and a systematic evaluation of their diagnostic performance across different clinical contexts is lacking [22,23,24,25].

Despite the growing number of studies evaluating these biochemical indices, no systematic review has comprehensively compared their diagnostic performance or assessed the quality of evidence supporting their use. Furthermore, the applicability of these tools in primary care settings, where early identification and appropriate triage of hyperparathyroidism patients is crucial, has not been systematically evaluated. The objective of this review was to analyze the diagnostic utility of the Ca/P ratio, the PFindex, the WIN, the Cl/P ratio, the Ca × Cl/P quotient, and the TCT in the identification and differentiation of various forms of hyperparathyroidism compared with currently employed reference methods, with the purpose of determining their usefulness in settings such as primary care and providing evidence-based recommendations for their clinical application.

## 2. Materials and Methods

This systematic review followed the PRISMA (Preferred Reporting Items for Systematic reviews and Meta-Analyses, see Supplementary Materials) recommendations and was registered in the International Prospective Register of Systematic Reviews-PROSPERO with the number CRD420251152138.

### 2.1. Eligibility Criteria

Original studies evaluating the use of indices based on serum biochemical tests for the diagnosis of hyperparathyroidism were included. Reviews, case reports, animal studies, or those without sufficient data were excluded. For synthesis, studies were grouped according to the diagnostic tool analyzed.

### 2.2. Information Sources

Searches were conducted in PubMed/Medline, Scopus, Web of Science, and Scielo. Also, in the Cochrane Central Register of Controlled Trials (CENTRAL). The search was restricted to the last five years and publications in any language were considered. The last electronic search was performed on 25 January 2026.

### 2.3. Search Strategy

A systematic search strategy was designed with the collaboration of a biomedical information specialist, using a combination of controlled terms (MeSH, Emtree, DeCS) and free terms. Additionally, references of included articles were manually reviewed to identify additional studies.

The specific search formulas applied in each database are presented in Table 1.

### 2.4. Study Selection Process

All records retrieved from the databases were managed in Rayyan AI^®^ (Qatar Computing Research Institute, Doha, Qatar; available at: https://www.rayyan.ai; accessed 8 February 2026), where duplicates were automatically removed. Subsequently, three authors independently screened the titles and abstracts of each record against the predefined inclusion criteria. Studies considered potentially eligible were evaluated at full text. Disagreements were resolved through consensus.

### 2.5. Data Extraction Process

Three reviewers independently extracted the results, and these were compared to homogenize them and discuss differences. Consensus was always chosen as the method for resolving differences. In cases of incomplete or unreported information, attempts were made to contact the study authors to complete it.

### 2.6. Data List

Data were collected on the diagnostic accuracy of indices used to facilitate the diagnosis of hyperparathyroidism, including the Ca/P, Cl/P, CaCl/P ratios, the PFindex, the WIN, and the TCT. Values for area under the curve (AUC), sensitivity, specificity, and cutoff points were recorded.

### 2.7. Handling of Cut-Off Values and Diagnostic Reference Standard

Given the expected variability in cut-off values across studies, which is inherent to diagnostic accuracy research, we adopted the following approach: (1) we extracted and reported the optimal cut-off points identified in each individual study; (2) we documented the range of cut-off values for each index to characterize the extent of threshold variability; (3) we employed the hierarchical summary receiver operating characteristic (HSROC) model for meta-analysis, which explicitly accounts for the expected negative correlation between sensitivity and specificity as cut-off points vary between studies, providing more robust estimates than models that treat these parameters independently.

The diagnostic reference standard for primary hyperparathyroidism was defined according to current clinical guidelines: persistent hypercalcemia (or normocalcemia in NHPT) with elevated or inappropriately normal PTH levels, after exclusion of secondary causes including vitamin D deficiency, chronic kidney disease, and medication effects. Studies were required to clearly define their diagnostic criteria for inclusion. For studies comparing PHPT with secondary hyperparathyroidism, the reference standard for SHPT included documented secondary causes such as vitamin D deficiency or chronic kidney disease with appropriate biochemical profiles [19,20,21,22,23,24,25].

### 2.8. Risk of Bias Assessment of Individual Studies

Risk of bias was assessed using specific tools according to the methodological design of each investigation, such as the QUADAS-2 tool (Quality Assessment of Diagnostic Accuracy Studies), the Newcastle-Ottawa Scale (NOS), and STROBE (Strengthening the Reporting of Observational Studies in Epidemiology). Three reviewers performed the assessment independently, and disagreements were resolved through discussion and consensus.

### 2.9. Effect Measures

For each outcome, the measures presented in the articles themselves were collected and presented. In studies that reported multiple subgroups, these measures were presented by subgroup. No additional data transformations were performed, except for conversion of measurement units; results were synthesized and compared directly using the original metrics from each study.

### 2.10. Synthesis Methods

The initial synthesis was narrative, although meta-analyses were performed for quantitative synthesis, employing a hierarchical bivariate random-effects model (HSROC model—Hierarchical Summary Receiver Operating Characteristics) for indices evaluated in two or more studies. This model explicitly considers the expected negative correlation between sensitivity and specificity as cutoff points vary between studies, providing more robust and less biased estimates than independent models. For each index, pooled estimates of sensitivity and specificity, the Diagnostic Odds Ratio (DOR), and the area under the SROC curve (AUC-SROC), with their respective 95% confidence intervals, were calculated. Heterogeneity was assessed using the I^2^ statistic for each parameter.

To address clinical heterogeneity, we planned a priori to conduct subgroup analyses when sufficient data were available, stratifying by (1) hyperparathyroidism subtype (hypercalcemic PHPT vs. NHPT); (2) control group type (healthy controls vs. secondary hyperparathyroidism); (3) geographic region. However, the limited number of studies reporting stratified data precluded formal subgroup meta-analyses for most indices. Instead, we narratively synthesized the available evidence on index performance across different clinical subgroups and explicitly reported the limitations imposed by clinical heterogeneity. The decision to pool data was based on both clinical judgment regarding the similarity of study populations and methodological assessment, rather than relying solely on statistical tests of heterogeneity

### 2.11. Publication Bias Assessment and Certainty of Evidence Assessment

The risk of publication bias was assessed through literature review and analysis of possible missing results, using formal statistical tests such as funnel plots. The certainty of evidence for each outcome was assessed following GRADE (Grading of Recommendations Assessment, Development and Evaluation) principles, considering risk of bias, inconsistency, imprecision, indirectness, and publication bias. Each outcome was classified into confidence levels (high, moderate, low, or very low).

## 3. Results

### 3.1. Search Results and Study Selection Process

The systematic search identified a total of 879 potentially relevant records. After removing 114 duplicates, 765 titles and abstracts were screened. The majority of records were excluded for not meeting eligibility criteria.

**Figure 1:**
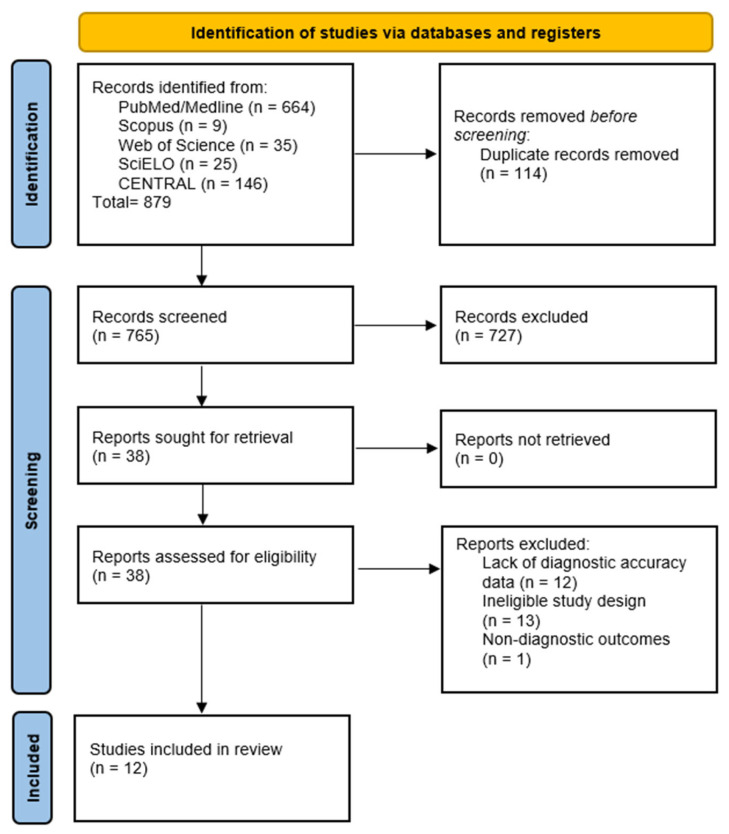
PRISMA flowchart summarizing the manuscript screening strategy.

Thirty-eight articles were evaluated at full text. Of these, 26 were excluded due to absence of sensitivity, specificity, or ROC curve data, for employing ineligible designs, or for focusing on non-diagnostic outcomes. Finally, 12 studies met the inclusion criteria. The selection process is summarized in the PRISMA flow diagram (see Figure 1).

### 3.2. General Characteristics of Included Studies

The 12 included studies were published between 2020 and 2025 and were conducted in Europe, Asia, and North America, specifically in Italy, China, the United States, Turkey, Romania, and Belgium. Regarding design, the majority corresponded to retrospective case–control studies [23,26,27,28,29,30,31], followed by retrospective observational studies [32,33,34] and one multicenter cross-sectional study [22]. A single study evaluated a dynamic test using a retrospective cohort design [35].

Sample size ranged from 20 to more than 800 participants. Studies included patients with hypercalcemic primary hyperparathyroidism, normocalcemic primary hyperparathyroidism, secondary hyperparathyroidism, and healthy controls. Several studies evaluated more than one diagnostic index simultaneously.

### 3.3. Risk of Bias of Individual Studies

Diagnostic accuracy studies demonstrated a predominantly low overall risk, due to the use of valid reference standards and objective measurements of biochemical indices. In contrast, case–control studies presented mostly moderate risk, attributable to limitations in the selection and comparability of control groups. Two studies exhibited high risk due to fundamental methodological problems in their designs. Overall, the applicability of findings is considered acceptable (See Table 2).

### 3.4. Results by Diagnostic Tool

#### 3.4.1. Calcium/Phosphorus Ratio (Ca/P)

The Ca/P ratio was evaluated in seven studies, being the most frequently analyzed index [23,26,27,28,29,30,32]. Consistently, patients with primary hyperparathyroidism presented significantly higher Ca/P values compared to healthy controls and other types of hyperparathyroidism. In the multicenter study by Madeo et al. [26], the Ca/P ratio allowed discrimination of primary hyperparathyroidism from controls with a sensitivity of 88.2% and specificity of 87.9% for a cutoff of 2.55 mmol/L. Guo et al. [27] observed a progressive gradient of Ca/P values from healthy subjects and patients with secondary hyperparathyroidism to normocalcemic and primary hyperparathyroidism, with a sensitivity of 91.4% and specificity of 93.9% for a cutoff of 2.71 mmol/L. Wright et al. [28] reported high sensitivity (95.6%) although with moderate specificity (63.6%), while Yin et al. [29] described exceptional performance with an AUC of 0.993 (sensitivity of 95.5% and specificity of 98.7% for a cutoff of 2.94).

For their part, Bestepe et al. [30] confirmed the utility of the index in both hypercalcemic and normocalcemic primary hyperparathyroidism, albeit with lower sensitivity in the latter subgroup. Özkan et al. [23] and Castellano et al. [32] showed that the Ca/P ratio maintains diagnostic utility, but with inferior performance to indices that incorporate PTH.

#### 3.4.2. Parathyroid Function Index (PFindex: Ca × PTH/P)

The PFindex was evaluated in two studies [23,27]. In both, the PFindex showed significantly higher values in patients with primary hyperparathyroidism compared to controls and secondary hyperparathyroidism.

Guo et al. [27] reported a sensitivity of 96.9% and specificity of 97.6% for a cutoff > 34, being the index with the highest diagnostic performance among those evaluated in that study. Özkan et al. [23] observed that the PFindex surpassed WIN and the Ca/P ratio in the diagnosis of normocalcemic primary hyperparathyroidism, with an AUC of 0.932. However, it is important to note that these findings are based on only two studies with a combined total of 439 patients, which limits the generalizability of these results. External validation in larger, independent cohorts is essential before definitive conclusions can be drawn about the superiority of PFindex over other indices.

#### 3.4.3. Wisconsin Index (WIN: Ca × PTH)

WIN was also evaluated in two studies [23,27]; in both, significantly higher index values were evident in patients with primary hyperparathyroidism. In the study by Guo et al. [27], WIN achieved a sensitivity of 93.0% and specificity of 97.6%. However, Özkan et al. [23] observed a decrease in diagnostic performance in the context of normocalcemic hyperparathyroidism, being inferior to PFindex.

#### 3.4.4. Chloride/Phosphorus Ratio (Cl/P)

The Cl/P ratio was evaluated in three studies [28,29,31]. Results showed considerable heterogeneity. For example, Wright et al. [28] reported low diagnostic performance with limited specificity. In contrast, Yin et al. [29] described an AUC of 0.966 with high sensitivities and specificities, and for their part, Yu et al. [31] observed intermediate performance, with high sensitivity but moderate specificity.

#### 3.4.5. Ca × Cl/P Ratio

The Ca × Cl/P quotient was evaluated in the study by Yu et al. [31]. This index showed high diagnostic performance for the diagnosis of primary hyperparathyroidism, with an AUC of 0.964 and sensitivity and specificity values above 90%. In this study, the Ca × Cl/P quotient surpassed the Ca/P ratio and the Cl/P ratio.

Given that this index was evaluated in a single retrospective study, these results should be interpreted with caution. Independent validation in prospective, multicenter studies is required before recommending its adoption in clinical practice.

#### 3.4.6. Thiazide Challenge Test (TCT)

The thiazide challenge test was evaluated in the study by Verly et al. [35]. This test allowed differentiation of primary hyperparathyroidism from secondary hyperparathyroidism associated with idiopathic hypercalciuria with a sensitivity of 81.8% and specificity of 77.8%, albeit with a relevant rate of inconclusive results and misclassification.

### 3.5. Synthesis of Results

The synthesis of the 12 included studies reveals clear differences in the diagnostic performance of different tools based on serum biomarkers, both in terms of sensitivity and specificity and stability across populations and hyperparathyroidism subtypes. Although several manuscripts evaluated more than one index simultaneously, the results allow identification of consistent patterns in the performance of each tool.

The Ca/P ratio was evaluated in seven manuscripts, with a cumulative number of participants exceeding 2800 subjects, including patients with primary hyperparathyroidism (hypercalcemic and normocalcemic), other forms of hyperparathyroidism, and healthy controls. The optimal cutoff points identified for the diagnosis of primary hyperparathyroidism were situated in a relatively narrow range, between 2.28 and 2.94 mmol/L. Reported sensitivity ranged between 88.2% and 95.6%, while specificity showed greater variability, with values between 63.6% and 98.7% [23,26,27,28,29,30,32].

The PFindex was evaluated in two studies, with a combined total of 439 patients, including subjects with hypercalcemic and normocalcemic primary and secondary hyperparathyroidism [23,27]. In both studies, the PFindex showed superior diagnostic performance to indices that do not incorporate PTH. In quantitative terms, the PFindex showed less overlap of values between diagnostic groups, which translated into fewer false positives and false negatives compared to simple indices [23,27].

WIN was evaluated in two studies, with populations similar to those used for PFindex [23,27]. However, WIN performance was inferior to PFindex when both tools were directly compared. These data indicate that, although WIN presents good discriminative capacity, its performance is more variable and dependent on the hyperparathyroidism subtype [23,27]. For its part, the Cl/P ratio was evaluated in three studies, with heterogeneous results, evidencing substantial variability with absolute differences of more than 60 percentage points in specificity between studies [28,29,31].

Regarding the Ca × Cl/P quotient, this was evaluated in a single study with a total sample of 460 participants and showed an AUC of 0.964 [31]. Comparatively, the Ca × Cl/P quotient surpassed the Ca/P ratio (AUC 0.956), the Cl/P ratio (AUC 0.923), and albumin-corrected calcium (AUC 0.959) in the same population. Additionally, it maintained good performance in both hypercalcemic and normocalcemic primary hyperparathyroidism patients, with a sensitivity of 83.1% and specificity of 93.8% in the latter subgroup [31].

Table 3 presents a summary of the studies with their included population data, sensitivity, specificity, and cutoff point.

### 3.6. Meta-Analysis

Meta-analyses were performed using the HSROC model for indices evaluated in two or more studies: Ca/P ratio (7 studies), PFindex (2 studies), WIN (2 studies), and Cl/P ratio (3 studies). Pooled performance metrics such as sensitivity, specificity, DOR, and AUC-SROC, along with the degree of heterogeneity (I^2^) and main conclusions for each biomarker, are presented in Table 4.

**Figure 2:**
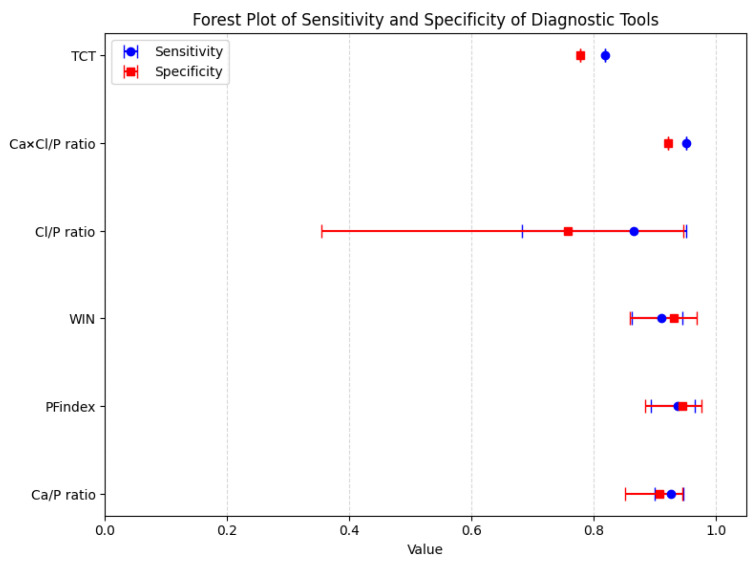
Forest Plot summarizing the pooled estimates for each diagnostic tool. Ca/P, calcium-to-phosphorus ratio; PFindex, Parathyroid Function Index; WIN, Wisconsin Index; Cl/P, chloride-to-phosphorus ratio; Ca × Cl/P, calcium-chloride-to-phosphorus ratio; TCT, Thiazide Challenge Test; CI, confidence interval; Sens, sensitivity; Spec, specificity.

The Forest Plot in Figure 2 summarizes the pooled estimates for each diagnostic tool. The sensitivity and specificity of each tool are presented with 95% confidence intervals, allowing visual comparison of their diagnostic accuracy and consistency across studies. Tools such as Ca/P, PFindex, and WIN show high and consistent performance, while others, such as Cl/P, present greater heterogeneity and more variable and imprecise clinical utility.

**Figure 3:**
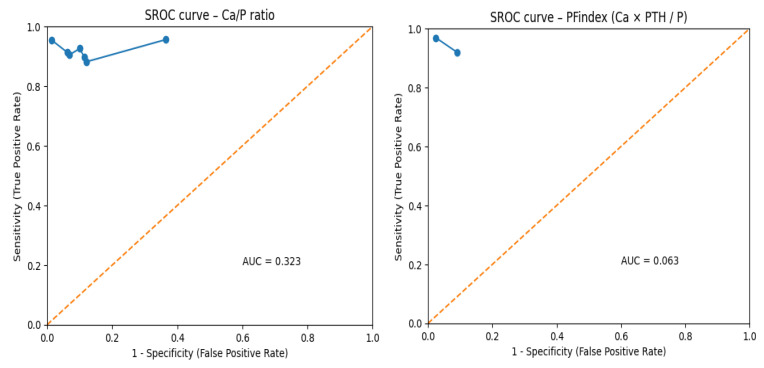

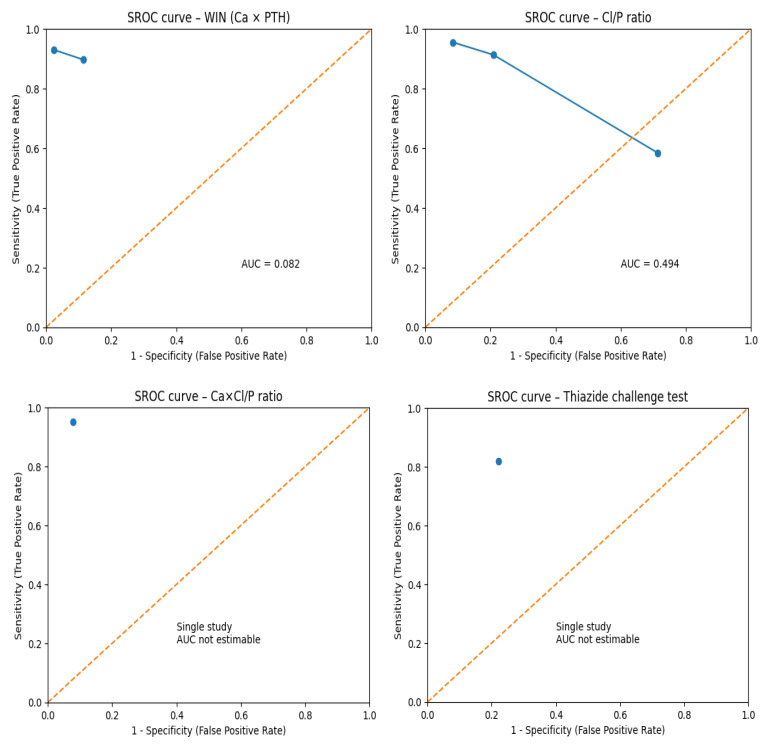
AUC-SROC for the different indices, presented individually. AUC-SROC, area under the summary receiver operating characteristic curve; Ca/P, calcium-to-phosphorus ratio; PFindex, Parathyroid Function Index; WIN, Wisconsin Index; Cl/P, chloride-to-phosphorus ratio.

**Figure 4:**
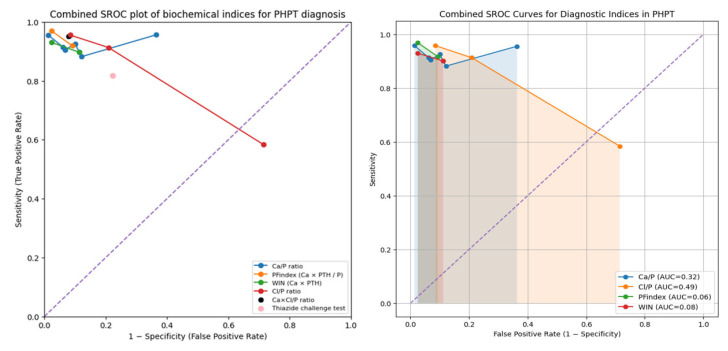
Pooled AUC-SROC combining the different indices allowing direct comparison. AUC-SROC, area under the summary receiver operating characteristic curve; Ca/P, calcium-to-phosphorus ratio; PFindex, Parathyroid Function Index; WIN, Wisconsin Index; Cl/P, chloride-to-phosphorus ratio.

In the ROC space, each point represents an individual study, and for indices with multiple studies, the SROC curve corresponding to their hierarchical model was plotted (Figure 3). Figure 4 superimposes all estimated SROC curves in a single graph, allowing direct comparison of their diagnostic performance.

The SROC curves reveal clear differences between indices. Both the Ca/P ratio and PFindex present curves close to the upper left corner (optimal precision zone), with areas under the curve of 0.957 and 0.943, respectively. This corroborates their excellent discriminative capacity and their robustness as diagnostic tools. The WIN index also shows good performance (AUC-SROC = 0.922), slightly inferior to PFindex but overlapping with Ca/P in its region of greatest clinical utility.

In marked contrast, the SROC curve of the Cl/P ratio is notably situated closer to the line of no discrimination (AUC-SROC = 0.812), confirming its moderate and inconsistent diagnostic performance, with low pooled specificity (0.634) that limits its clinical applicability. The Ca × Cl/P index and the thiazide challenge test, despite showing promising results in single studies (with reported sensitivities of 0.95 and 0.82, and specificities of 0.92 and 0.78, respectively), lack sufficient evidence to draw generalizable conclusions. Their validation in independent and larger cohorts is a priority for future research.

#### Performance Across Clinical Subgroups

Although formal subgroup meta-analyses were not feasible due to limited stratified reporting, narrative synthesis of available data revealed important patterns across clinical subgroups. For PHPT versus healthy controls, all indices demonstrated excellent performance, with the Ca/P ratio showing sensitivities consistently above 88% and specificities above 85% across studies [23,26,27,28,29,30]. However, when distinguishing PHPT from secondary hyperparathyroidism, performance was more variable; the PFindex showed superior discrimination in this context compared to indices not incorporating PTH [23,24,25,26,27].

For NHPT, all indices exhibited substantially reduced diagnostic accuracy. Bestepe et al. [30] reported that the Ca/P ratio maintained utility in NHPT but with lower sensitivity compared to hypercalcemic presentations. More critically, recent evidence suggests that biochemical ratios including Ca/P and Ca × Cl/P show high sensitivity but poor specificity (ranging from 1.6% to 23.2%) when attempting to distinguish NHPT from vitamin D deficiency-related secondary hyperparathyroidism, as these conditions share similar biochemical profiles. This finding underscores that current biochemical indices cannot reliably differentiate NHPT from vitamin D deficiency-related secondary hyperparathyroidism, and rigorous exclusion of secondary causes remains essential before establishing a diagnosis of normocalcemic primary hyperparathyroidism [23,26,27].

Regarding cut-off variability, the Ca/P ratio cut-offs ranged from 2.28 to 2.94 mmol/L across studies, reflecting differences in study populations, laboratory methods, and optimization strategies. Despite this variability, the HSROC model accounts for threshold effects, and the pooled estimates represent average diagnostic performance across the range of clinically applied cut-offs. The most commonly identified optimal cut-off was 2.55 mmol/L, reported in three independent studies [23,26,27].

### 3.7. Certainty of Evidence

Evidence assessment using GRADE revealed differences between diagnostic indices. Ca/P and PFindex showed moderate certainty, although their reliability could change with future studies, mainly due to biases derived from retrospective designs and post hoc defined cutoff points. The WIN index presented moderate certainty, limited by few studies and small sample sizes, despite consistent results. Cl/P and Ca × Cl/P showed low certainty due to bias, inconsistency, and imprecision. The thiazide test presented very low certainty, reflecting serious biases and great imprecision, which considerably reduces confidence in its results. See Table 5.

**Figure 5:**
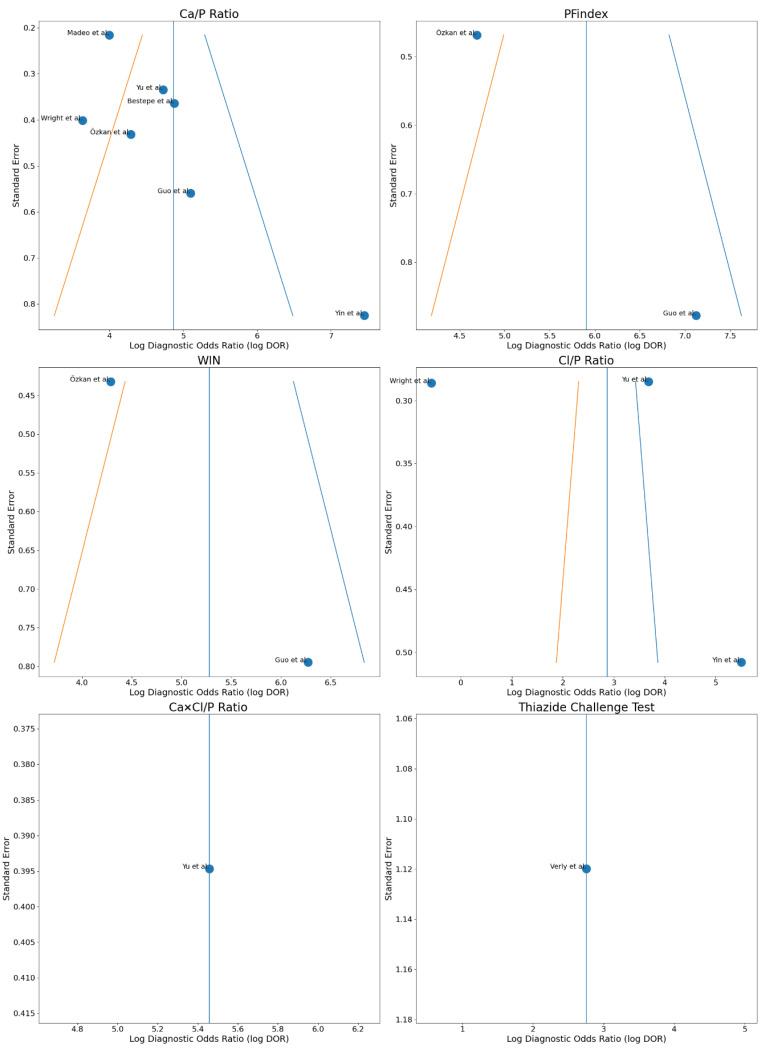
Funnel plot for the assessment of publication bias of the different diagnostic indices in primary hyperparathyroidism [23,26,27,28,29,30,31,35]. DOR, diagnostic odds ratio; SE, standard error; Ca/P, calcium-to-phosphorus ratio; PFindex, Parathyroid Function Index; WIN, Wisconsin Index; Cl/P, chloride-to-phosphorus ratio; Ca × Cl/P, calcium-chloride-to-phosphorus ratio.

Visual assessment of classic funnel plots (log DOR versus standard error) in Figure 5 showed a globally symmetric distribution for most diagnostic indices, although some asymmetry was observed in the Ca/P ratio in studies with higher standard error. In statistical analysis, Egger’s regression test was significant for the Ca/P ratio (*p* = 0.003), suggesting the possible presence of publication bias or small-study effects, while Begg’s test was not significant (*p* = 0.136). For the Cl/P ratio (3 studies), neither test showed evidence of publication bias, although interpretation is limited by the small number of studies. Formal assessment of publication bias was not possible for the remaining indices due to the insufficient number of available studies, and the funnel plot should be considered an exploratory test.

## 4. Discussion

In this systematic review, the diagnostic utility of various serum biochemical indices for the diagnosis of hyperparathyroidism was evaluated, including the Ca/P ratio, PFindex, WIN, Cl/P ratio, Ca × Cl/P quotient, and the thiazide challenge test. The findings suggest that the Ca/P ratio and PFindex present the most favorable diagnostic performance among the evaluated indices, with pooled sensitivities and specificities exceeding 90% and SROC areas under the curve of 0.957 and 0.943, respectively. These results position both indices as valid and clinically useful screening tools for the diagnosis of primary hyperparathyroidism [23,26,27,28,29,30,31].

The Ca/P ratio has demonstrated notable consistency across multiple studies and populations. With seven studies including more than 2900 participants, this index showed a pooled sensitivity of 91.6% and specificity of 89.3% [23,26,27,28,29,30,31]. The homogeneity in the identified cutoff points (2.28–2.94 mmol/L) suggests that this index is robust and reproducible in different clinical contexts. However, it is important to highlight that the moderate-high heterogeneity observed in specificity (I^2^ = 92.7%) reflects variations in the populations studied and in the control selection criteria. This variability underscores the need to consider the clinical context when interpreting results, particularly in patients with conditions affecting calcium–phosphorus metabolism, such as vitamin D deficiency or chronic renal insufficiency [36,37,38].

For its part, the PFindex, which incorporates PTH in its formula (Ca × PTH/P), showed superior diagnostic performance in studies that evaluated it directly against other indices. With a pooled sensitivity of 94.4% and specificity of 94.2%, the PFindex consistently surpassed the Ca/P ratio and WIN in discriminating between primary and secondary hyperparathyroidism [23,27]. The incorporation of PTH in the index calculation appears to confer a significant diagnostic advantage, as it more directly reflects the autonomous parathyroid function characteristic of primary hyperparathyroidism. Additional studies have demonstrated that the PFindex correlates strongly with PTH and parathyroid adenoma size, suggesting that it is not only a diagnostic marker but also an indicator of disease severity [33,39]. Critically, the available evidence is limited to only two studies with 439 patients, which substantially limits the strength of conclusions that can be drawn. Although the GRADE rating indicates moderate certainty, this reflects the consistency of findings within the available studies rather than the robustness of the overall evidence base. The apparent superiority of PFindex over other indices should therefore be interpreted cautiously, and validation in larger, prospective, multicenter cohorts is essential before recommending its preferential use over more widely studied indices such as the Ca/P ratio.

WIN, although conceptually similar to PFindex, showed slightly inferior performance, with a pooled sensitivity of 91.3% and specificity of 93.1% [23,27]. Originally designed to predict the probability of multiglandular disease during parathyroidectomy, WIN has been explored as a diagnostic tool with variable results. The moderate heterogeneity observed (I^2^ = 36.3% for sensitivity) suggests that its performance may be more dependent on the hyperparathyroidism subtype and population characteristics. Subsequent studies have questioned its utility without the use of additional intraoperative studies such as intraoperative PTH, reporting adenoma cure failure rates of up to 13% when used in isolation [40,41,42].

The Cl/P ratio presented the most variable and least consistent performance among all indices evaluated. With a pooled specificity of only 63.4% and very high heterogeneity (I^2^ = 98.8%), this index showed significant limitations for generalized clinical application [28,29,31]. The extreme variability in results between studies (specificity from 28.6% to 91.5%) suggests that methodological, population, or laboratory factors may substantially influence its performance. Although some studies have reported utility when combined with alkaline phosphatase, particularly in Asian populations, current evidence does not support its routine use as an independent diagnostic tool [28,43,44].

The Ca × Cl/P quotient emerged as a promising index in the only study that evaluated it, showing an AUC greater than 0.9 and surpassing the Ca/P ratio, Cl/P, and albumin-corrected calcium in the same population [31]. Particularly notable was its performance in normocalcemic primary hyperparathyroidism, with sensitivity of 83.1% and specificity of 93.8%. However, the evidence is limited to a single retrospective study with 460 participants, resulting in low certainty of evidence according to GRADE. External validation in independent and prospective cohorts is essential before recommending its adoption in clinical practice [31,45,46].

Normocalcemic primary hyperparathyroidism represents a particular diagnostic challenge. The results of this review demonstrate that all biochemical indices show significant limitations in this context, with specificities ranging between 1.6% and 23.2%. This low specificity reflects the biochemical overlap between normocalcemic hyperparathyroidism and secondary hyperparathyroidism related to vitamin D deficiency, conditions that may be indistinguishable based solely on static biochemical parameters. Rigorous exclusion of secondary causes of PTH elevation, including vitamin D deficiency, chronic renal insufficiency, idiopathic hypercalciuria, and use of medications such as lithium or thiazides, is fundamental before establishing the diagnosis of normocalcemic hyperparathyroidism [36,39,47,48].

When serum biomarker-based indices fail to establish a definitive diagnosis, particularly in normocalcemic presentations or when biochemical results are equivocal, clinicians should adopt a systematic diagnostic approach. First, repeated measurements of calcium (both total and ionized) and PTH are essential, as patients with PHPT may occasionally have normal calcium levels but are hypercalcemic most of the time. Second, rigorous exclusion of secondary causes is mandatory: vitamin D deficiency should be corrected and biochemical evaluation repeated after 6–8 weeks; medications that may mimic PHPT (thiazides, lithium) should be discontinued when possible; and renal function should be assessed to exclude chronic kidney disease. Third, a 24 h urinary calcium measurement is important to distinguish PHPT from familial hypocalciuric hypercalcemia, which can mimic PHPT biochemically. Fourth, when biochemical diagnosis remains uncertain, serial biochemical monitoring over time may reveal the dynamic nature of the disease, as recent evidence suggests that integrating serial biochemical measurements with targeted imaging can facilitate earlier diagnosis [49,50,51].

Finally, the thiazide challenge test was evaluated in a single small study with 20 patients, showing sensitivity of 81.8% and specificity of 77.8% for differentiating primary hyperparathyroidism from secondary hyperparathyroidism associated with idiopathic hypercalciuria [35]. Although conceptually attractive, the TCT presented important limitations, including a relevant rate of inconclusive results and misclassification. The certainty of evidence was rated as very low due to serious biases, imprecision, and the reduced sample size. Previous studies have demonstrated that thiazides may be safe in patients with primary hyperparathyroidism for hypercalciuria control, but require careful monitoring of calcemia. The utility of TCT as a diagnostic tool requires validation in larger prospective studies before its routine use can be recommended [49,50,51]. Given the very limited evidence (single study, 20 patients, very low GRADE certainty), no clinical recommendations can be made regarding the TCT at this time. This test should be considered experimental and requires substantial additional research before any conclusions about its clinical utility can be drawn.

An important finding of this review is the evidence of publication bias for the Ca/P ratio, detected by Egger’s regression test (*p* = 0.003). This suggests that studies with negative or less favorable results may not have been published, which could overestimate the true diagnostic performance of this index. This finding has important implications for the interpretation of our pooled estimates: the reported sensitivity of 91.6% and specificity of 89.3% for the Ca/P ratio may represent upper-bound estimates rather than true population values. Clinicians should therefore apply these indices with appropriate caution, recognizing that real-world performance may be somewhat lower than suggested by the pooled meta-analytic estimates. Although the large number of studies and overall consistency of results provide some reassurance, the potential for overestimation should be explicitly acknowledged when applying these findings to clinical practice.

The strengths of this review include the exhaustive systematic search across multiple databases, rigorous risk of bias assessment using validated tools, and the performance of meta-analyses with hierarchical bivariate models that appropriately consider the correlation between sensitivity and specificity. The inclusion of studies published in the last five years ensures that the findings reflect contemporary clinical practice.

Nevertheless, several important limitations must be acknowledged. First, clinical heterogeneity represents a major limitation of this review. The combined analysis of heterogeneous populations, including PHPT, NHPT, SHPT, and healthy controls, limits the interpretability of pooled diagnostic accuracy measures. Although we recognized the diagnostic challenges across these subgroups, the limited availability of stratified data in primary studies precluded systematic subgroup analyses. Future primary studies should report diagnostic performance separately for each clinical subgroup to enable more precise meta-analytic estimates.

Second, the substantial variability in cut-off values across studies (e.g., Ca/P ratio cut-offs ranging from 2.28 to 2.94 mmol/L) reflects the context-dependent nature of diagnostic performance and highlights the need for standardization. While the HSROC model accounts for threshold effects statistically, clinicians should recognize that optimal cut-offs may vary depending on local laboratory methods, population characteristics, and the specific clinical question (screening vs. confirmation).

Third, the majority of included studies were retrospective, with risk of selection bias and post hoc defined cutoff points, which may overestimate diagnostic performance. This methodological limitation deserves particular emphasis. Data-driven selection of optimal cutoff values has been shown to introduce positive bias in diagnostic accuracy estimates, with studies demonstrating that post hoc optimization can exaggerate sensitivity and specificity by approximately 5–6 percentage points in studies with fewer than 100 diseased subjects. The magnitude of this bias is inversely related to sample size; thus, smaller studies in our meta-analysis may contribute disproportionately inflated estimates. Furthermore, retrospective designs are associated with higher estimates of diagnostic accuracy compared to prospective studies, with relative diagnostic odds ratios of approximately 1.6 favoring retrospective data collection. These methodological considerations suggest that the pooled diagnostic performance reported in this review should be considered optimistic estimates that require prospective validation [52,53,54].

Fourth, evidence for some indices (PFindex, WIN, Ca × Cl/P, TCT) is based on a limited number of studies, which reduces confidence in pooled estimates.

The clinical implications of these findings are significant. The Ca/P ratio emerges as a simple, economical, and widely available screening tool that can facilitate early identification of primary hyperparathyroidism, particularly in resource-limited settings or in primary care. A cutoff point of 2.55 mmol/L (3.3 if expressed in mg/dL) demonstrated an optimal balance between sensitivity and specificity. The PFindex, although requiring PTH measurement, offers superior discrimination between primary and secondary hyperparathyroidism, which may be particularly useful in ambiguous cases or when differentiation is critical for therapeutic decisions. However, it is essential to emphasize that no biochemical index should be used in isolation; integration with clinical evaluation, exclusion of secondary causes, and, when indicated, imaging studies remain essential for definitive diagnosis.

Future research directions should include prospective multicenter studies that validate these indices in diverse populations, including different ethnic groups and geographic contexts. Standardization of laboratory methods and cutoff points is crucial to improve reproducibility. Finally, evaluation of the cost-effectiveness of incorporating these indices into diagnostic algorithms compared with conventional strategies could inform clinical practice guidelines and optimize resource utilization.

## 5. Conclusions

This systematic review demonstrates that the Ca/P ratio and PFindex are valid and clinically useful diagnostic tools for the diagnosis of hypercalcemic primary hyperparathyroidism that could be utilized in primary care because they only require basic laboratory testing. However, several important caveats must be emphasized. First, the evidence for PFindex is based on only two studies, and the Ca × Cl/P ratio on a single study, limiting the strength of conclusions regarding these indices. Second, evidence of publication bias for the Ca/P ratio suggests that pooled estimates may be optimistic. Third, and most critically, all indices show very poor specificity (1.6–23.2%) in the diagnosis of normocalcemic hyperparathyroidism, rendering them unreliable for distinguishing NHPT from secondary hyperparathyroidism related to vitamin D deficiency. These biochemical indices should therefore not be used in isolation for NHPT; rigorous exclusion of secondary causes, repeated biochemical measurements, and, when surgery is contemplated, advanced imaging modalities remain essential components of the diagnostic approach in this challenging clinical context.

## Figures and Tables

**Table 1:** Search formulas in the different databases consulted.

Database	Search Formula
PubMed/MEDLINE	(“Hyperparathyroidism”[Mesh] OR “Hyperparathyroidism, Primary”[Mesh] OR “Hyperparathyroidism, Secondary”[Mesh] OR hyperparathyroid[tiab] OR phpt[tiab] OR nhpt[tiab]) AND (“Diagnostic Tests, Routine”[Mesh] OR “Sensitivity and Specificity”[Mesh] OR “ROC Curve”[Mesh] OR “diagnostic accuracy”[tiab] OR “differential diagnosis”[tiab] OR discriminat[tiab] OR “Biomarkers”[Mesh] OR index[tiab] OR indices[tiab] OR ratio[tiab] OR “wisconsin index”[tiab] OR “parathyroid function index”[tiab] OR pfindex[tiab] OR “calcium phosphate ratio”[tiab] OR “Challeng* test”[tiab] OR “thiazide challenge”[tiab] OR “tct test”[tiab] OR “provocat* test”[tiab])
Scopus	(TITLE (hyperparathyroidism) OR TITLE (hyperparathyroidism primary) OR TITLE (hyperparathyroidism secondary) AND TITLE (sensitivity) OR TITLE (specificity) OR TITLE (roc curve) OR TITLE (wisconsin INDEX) OR TITLE (parathyroid function INDEX) OR TITLE (pfindex) OR TITLE (calcium phosphate ratio) OR TITLE (thiazide test)) AND PUBYEAR > 2019
Web of Science	((TI = (Hyperparathyroidism) OR TI = (“Hyperparathyroidism, Primary”) OR TI = (“Hyperparathyroidism, Secondary”) OR TI = (hyperparathyroid)) AND (TI = (“Diagnostic Tests, Routine”) OR TI = (“Sensitivity and Specificity”) OR TI = (“ROC Curve”) OR TI = (“diagnostic accuracy”) OR TI = (Biomarkers) OR TI = (index) OR TI = (indices) OR TI = (ratio) OR TI = (“wisconsin index”) OR TI = (“parathyroid function index”) OR TI = (pfanner) OR TI = (“calcium phosphate ratio”) OR TI = (“thiazide challenge”)))
SciELO	(tw:(hiperparatiroidismo OR hyperparathyroidism OR “hiperparatireoidismo”) OR tw:(phpt OR nhpt)) AND (tw:(“exactitud diagnóstica” OR “diagnostic accuracy” OR “acurácia diagnóstica” OR “sensibilidad y especificidad” OR “sensitivity and specificity” OR “roc curve” OR “diagnóstico diferencial” OR “differential diagnosis”) OR tw:(índice OR index OR índice OR indices OR ratio OR razón OR proporção) OR tw:(“wisconsin index” OR “parathyroid function index” OR “índice de función paratiroidea” OR “índice de função paratireoidiana” OR pfindex) OR tw:(“calcium phosphate ratio” OR “relación calcio fósforo” OR “relação cálcio fósforo”) OR tw:(“prueba de provocación” OR “test de provocação” OR “challenge test” OR “thiazide challenge” OR “tct test”))
Cochrane CENTRAL	(hyperparathyroid* OR phpt OR nhpt) AND (“diagnostic accuracy” OR sensitivity OR specificity OR “roc curve” OR discriminant* OR index OR indices OR ratio OR “wisconsin index” OR “parathyroid function index” OR pfindex OR “calcium phosphate” OR “challenge test” OR “thiazide challenge” OR “tct test”)

The asterisk (*) is not an explanatory note but a truncation symbol used in the search strategy. It indicates that additional characters may follow the root of the word, allowing retrieval of all terms sharing the same word stem.

**Table 2:** Risk of bias assessment according to QUADAS-2, NOS, and STROBE.

Author, Year	Study Design	Assessment Tool	Domains Assessed (Score/Stars)	Global Risk	Main Justification
Madeo et al., 2020 [26]	Diagnostic accuracy study (multicenter retrospective cross-sectional)	QUADAS 2	Patient Selection: Low Index Test: Low Reference Standard: Low Flow & Timing: Low	LOW	Clear criteria, valid reference standard per guidelines, STARD diagram, large sample.
Guo et al., 2020 [27]	Retrospective case–control study	NOS (case–control)	Selection: ★★★☆ (3/4) Comparability: ★★ (2/2) Exposure: ★★☆ (2/3)	MODERATE	Cases from a single hospital, community controls, age-matched but not sex-matched.
Wright et al., 2020 [28]	Diagnostic accuracy study (retrospective cross-sectional)	QUADAS 2	Patient Selection: Moderate Index Test: Low Reference Standard: Low Flow & Timing: Low	MODERATE	Controls are thyroidectomized patients, limiting applicability to the general population.
Yin et al., 2021 [29]	Retrospective case–control study	NOS (case–control)	Selection: ★★★☆ (3/4) Comparability: ★☆ (1/2) Exposure: ★★☆ (2/3)	MODERATE	Controls from a check-up center (may be healthier), not matched, similar measurement.
Bestepe et al., 2022 [30]	Retrospective case–control study	NOS (case–control)	Selection: ★★★☆ (3/4) Comparability: ★☆ (1/2) Exposure: ★★☆ (2/3)	MODERATE	Historical controls from the same center, sex differences not controlled for in the design.
Özkan & Turhan, 2022 [23]	Retrospective case–control study	NOS (case–control)	Selection: ★★☆☆ (2/4) Comparability: ★☆ (1/2) Exposure: ★★☆ (2/3)	HIGH	Definition of SHPT may include residual VitD deficiency, groups intrinsically different.
De Vincentis et al., 2023 [22]	Diagnostic accuracy study (retrospective cross-sectional)	QUADAS 2	Patient Selection: Low Index Test: Low Reference Standard: Low Flow & Timing: Low	LOW	Confirmed genetic diagnosis, clear criteria, design similar to study 1.
Castellano et al., 2023 [32]	Observational cross-sectional study/Case series	Adapted STROBE/Case series	Representativeness: Moderate Measurement: Low Confounding Control: High Outcomes: Moderate	HIGH	No control group, comparisons between subgroups with high residual confounding, non-blinded symptom assessment.
Kolcsar et al., 2024 [33]	Retrospective cohort study	NOS (cohort)	Selection: ★★☆☆ (2/4) Comparability: ★☆ (1/2) Outcome: ★★☆ (2/3)	MODERATE TO HIGH	Single-center cohort, groups (NPHPT/HPHPT) not comparable at baseline, retrospective measurement of fractures.
Yu et al., 2024 [31]	Retrospective case–control study	NOS (case–control)	Selection: ★★★★ (4/4) Comparability: ★★ (2/2) Exposure: ★★☆ (2/3)	LOW	Controls 1:1 matched by age and sex from a large database, robust design, objective measurement.
Kappauf et al., 2024 [34]	Diagnostic accuracy study (retrospective cohort)	QUADAS 2	Patient Selection: Low Index Test: Low Reference Standard: Low Flow & Timing: Low	LOW	Consecutive patients, objective reference standard (PTH), contemporary measurements.
Verly et al., 2024 [35]	Retrospective diagnostic accuracy study	QUADAS 2	Patient Selection: High Index Test: Moderate Reference Standard: High Flow & Timing: Moderate	HIGH	Small and selected sample, non-uniform and non-blinded reference standard, high risk of differential verification bias.

The symbols ★ and ☆ indicate the degree of compliance with the assessed criteria: filled stars (★) represent the criteria met, while empty stars (☆) indicate the remaining margin for compliance.

**Table 3:** Summary of article results.

Study	Country	N	PHPT	Control	TP	FN	FP	TN	Sensitivity	Specificity	Cut-Off Point
Ca/P ratio											
Madeo et al. (2020) [26]	Italy	821	432	389	381	51	47	342	0.882	0.879	2.55 mmol/L
Wright et al. (2020) [28]	USA	303	226	77	216	10	28	49	0.956	0.636	2.55 mmol/L
Guo et al. (2020) [27]	China	210	128	82	117	11	5	77	0.914	0.939	2.71 mmol/L
Yin et al. (2021) [29]	China	296	143	153	137	6	2	151	0.955	0.987	2.94 mmol/L
Bestepe et al. (2022) [30]	Turkey	610	462	148	418	44	10	138	0.905	0.932	2.59 mmol/L
Özkan et al. (2022) [23]	Turkey	229	121	108	109	12	12	96	0.897	0.886	2.55 mmol/L
Yu et al. (2024) [31]	China	460	230	230	213	17	23	207	0.926	0.900	2.28 mmol/L
PFindex (Ca × PTH/P)											
Guo et al. (2020) [27]	China	210	128	82	124	4	2	80	0.969	0.976	34
Özkan et al. (2022) [23]	Turkey	229	121	108	111	10	10	98	0.919	0.909	327.8
WIN (Ca × PTH)											
Guo et al. (2020) [27]	China	210	128	82	119	9	2	80	0.930	0.976	35.43
Özkan et al. (2022) [23]	Turkey	229	121	108	109	12	12	96	0.897	0.886	1040.9
Cl/P ratio											
Wright et al. (2020) [28]	USA	303	226	77	132	94	55	22	0.584	0.286	33 mmol/L
Yin et al. (2021) [29]	China	296	143	153	137	6	13	140	0.955	0.915	32.4 mmol/L
Yu et al. (2024) [31]	China	460	230	230	210	20	48	182	0.913	0.791	96.13 mmol/L
Ca × Cl/P ratio											
Yu et al. (2024) [31]	China	460	230	230	219	11	18	212	0.952	0.922	239.17
Thiazide Challenge Test (TCT)											
Verly et al. (2025) [35]	Belgium	20	11	9	9	2	2	7	0.818	0.778	No cut-off point

**Table 4:** Meta-analysis results on the diagnostic performance of serum biomarker-based indices for primary hyperparathyroidism.

Diagnostic Tool	Number of Studies	Total Patients (PHPT/Control)	Pooled Sensitivity (95% CI)	Pooled Specificity (95% CI)	Pooled DOR (95% CI)	AUC-SROC	Heterogeneity (I^2^)	Main Conclusion
Ca/P ratio	7	2929 (1742/1187)	0.916 (0.879–0.942)	0.893 (0.826–0.936)	98.7 (50.2–194.1)	0.957 (0.926–0.988)	Sens: 68.3%	Excellent diagnostic performance. Valid and widely studied screening tool. Moderate-high heterogeneity in specificity.
							Espec: 92.7%	
PFindex	2	439 (249/190)	0.944 (0.905–0.968)	0.942 (0.901–0.967)	258.8 (92.6–723.3) *	0.943 (0.907–0.979)	Sens: 51.4%	Favorable performance in available studies, but evidence is limited to only 2 studies.
							Espec: 63.4%	
WIN	2	439 (249/190)	0.913 (0.867–0.944)	0.931 (0.888–0.958)	141.8 (56.6–355.4) *	0.922 (0.880–0.964)	Sens: 36.3%	Good performance, similar to Ca/P ratio. Lower heterogeneity than PFindex but less precise.
							Espec: 72.7%	
Cl/P ratio	3	1059 (599/460)	0.867 (0.717–0.944)	0.634 (0.380–0.830)	12.4 (3.1–49.8)	0.812 (0.738–0.886)	Sens: 97.5%	Moderate performance with low specificity. High heterogeneity limits its general clinical utility.
							Espec: 98.8%	
Ca × Cl/P ratio	1	460 (230/230)	0.952	0.922	No calculable	0.937 †	Not applicable	Excellent performance in a single study. Requires external validation.
TCT	1	20 (11/9)	0.818	0.778	No calculable	Not calculable	Not applicable	Promising utility in a specific and complex scenario. Low evidence.

*: DOR calculated with random-effects model (not HSROC due to only 2 studies). †: AUC estimated from individual study (Yu et al., 2024) [31]. Note: For indices with ≥2 studies (Ca/P, PFindex, WIN, Cl/P), pooled estimates were calculated using the HSROC model. For indices with only 1 study (Ca × Cl/P, TCT), values represent single-study estimates and should be interpreted with caution due to the absence of external validation. DOR and AUC-SROC could not be calculated for single-study indices using hierarchical models.

**Table 5:** Certainty of evidence rating according to GRADE methodology.

Diagnostic Index	No. Studies	Risk of Bias	Inconsistency	Indirectness	Imprecision	Publication Bias	Overall Certainty (GRADE)
Ca/P ratio	7	Serious	Not serious	Not serious	Not serious	Probable	⊕⊕⊕◯ MODERATE
PFindex (Ca × PTH/P)	2	Serious	Not serious	Not serious	Not serious	Not assessable	⊕⊕⊕◯ MODERATE
WIN (Ca × PTH)	2	Serious	Not serious	Not serious	Not serious	Not assessable	⊕⊕⊕◯ MODERATE
Cl/P ratio	3	Serious	Serious	Not serious	Serious	Probable	⊕⊕◯◯ LOW
Ca × Cl/P ratio	1	Serious	Not applicable	Not serious	Very serious	Not assessable	⊕⊕◯◯ LOW
TCT	1	Very serious	Not applicable	Serious	Very serious	Not assessable	⊕◯◯◯ VERY LOW

⊕ indicates the portion fulfilled or completed; ◯ indicates the remaining portion or potential.

## Data Availability

No new data were created or analyzed in this study. Data sharing is not applicable to this article.

## Supplementary Materials

The following supporting information can be downloaded at: https://www.mdpi.com/article/10.3390/healthcare14081001/s1. PRISMA_2020_checklist. Ref. [55] is cited in the supplementary material file.

## Abbreviations

The following abbreviations are used in this manuscript:

AUC	Area Under the Curve
Ca/P	Calcium-to-Phosphorus Ratio
Cl/P	Chloride-to-Phosphorus Ratio
DOR	Diagnostic Odds Ratio
GRADE	Grading of Recommendations Assessment, Development and Evaluation
HSROC	Hierarchical Summary Receiver Operating Characteristic
NHPT	Normocalcemic Hyperparathyroidism
PFindex	Parathyroid Function Index
PHPT	Primary Hyperparathyroidism
PRISMA	Preferred Reporting Items for Systematic Reviews and Meta-Analyses
PTH	Parathyroid Hormone
SHPT	Secondary Hyperparathyroidism
SROC	Summary Receiver Operating Characteristic
TCT	Thiazide Challenge Test
THPT	Tertiary Hyperparathyroidism
WIN	Wisconsin Index
